# A text dataset of campaign speeches of the main tickets in the 2020 US presidential election

**DOI:** 10.1038/s41597-025-04681-x

**Published:** 2025-04-19

**Authors:** Ioannis Chalkiadakis, Louise Anglès d’Auriac, Gareth W. Peters, Divina Frau-Meigs

**Affiliations:** 1 Institut des Systèmes Complexes de Paris Îlede-France, CNRS, Paris, 75013 France; 2 ED 625 MAGIIE, Université Sorbonne Nouvelle, Paris, 75012 France; 3 Department of Statistics and Applied Probability, University of California, Santa Barbara, 93106 USA

**Keywords:** Politics, Scientific data, Institutions, Communication, Computational science

## Abstract

Unstructured text data have gained popularity in political science, owing to advancements in rigorous ‘text-as-data’ methods that allow extracting insights into election outcomes, candidates’ appeal to voters, ideologies and campaign strategies. Existing datasets on US presidential election campaign speeches are limited in size or source variation, and often contain speeches of different types (debates, rallies, official presidential events, e.g. inauguration), thus lacking consistency in their rhetorical content. The introduced dataset comprises the campaign speeches of the Democratic and Republican tickets for the 2020 US presidential election (1, 056 in total), covering the period between January 2019 and January 2021. Importantly, the dataset dictates specific criteria for the rhetorical structure of the speech ensuring consistency, critical for quantitative analysis. It has been carefully curated, yet only to the necessary extent to still be able to inform studies that require semantic or grammatical/syntactical structure. The provided corpus is hosted on Zenodo and GitHub under the CC BY-NC 4.0 license, and it aims to enhance timely studies on US presidential elections with high-quality text data.

## Background & Summary

In quantitative politics and elections research traditionally the focus has been on voting (e.g. voter turnout, choice), citizen welfare, public opinion, or institutions (e.g. electoral systems, citizen trust), using data from surveys or polls, financial, demographic, or market indices. It is only recently that the value of unstructured data, particularly text data, has started being systematically leveraged in advanced statistical models in quantitative political science and digital humanities. We wish to distinguish here between ‘text-as-data’ methods and text mining or natural language processing (NLP) computer science approaches, which have an extensive history and literature. Although the communities interact with each other, we understand ‘text-as-data’ methods^1^ as being driven principally by developing statistical models to facilitate or quantitatively assess a theory coming from a field such as political science, sociology, law, economics etc. The text-as-data approach emphasizes forming a hypothesis (e.g. in political science), and sourcing curated text data that have attributes specific to the hypothesis and would help formulate a statistical model or test tailored to address it. Hence, this creates a requirement for specialist textual data (e.g. records of speeches with editorial oversight), careful data collection and curation to address that specific hypothesis. On the other hand, natural language processing approaches usually focus on extracting as much statistical structure as possible from an exhaustive collection of text data and subsequently investigating whether certain hypotheses can be addressed. Hence, both require different assumptions and validation methods. It is precisely these particular requirements that necessitate the construction of carefully curated datasets, comprised of data that are compatible with specific measurement rules, as the present Data Descriptor details for the provided dataset.

Text-as-data methods significantly enhanced the quantitative political science tool set. Statistical models that were previously mostly applied on roll call data^2,3^ (i.e. votes on legislation bills), and newly developed models, started leveraging text and text-based data^4^ (e.g. political party manifestos^5^, parliamentary speeches^6^, text sentiment), as covariates or latent variables in a standalone fashion, or alongside voting data^7,8^. These modeling advances allowed political scientists to leverage the rich amount of statistical structure present in textual datasets. This was necessary to facilitate the inclusion of more factors of variation (e.g. policy development, institutional context) than may be captured by voting patterns alone^9^. Importantly, the development of models for text in such as way as to be approachable by non-STEM users, for instance, because they are based on classical statistical models that they are familiar with (e.g. Item-Response Theory), has extended the impact of text-as-data and quantitative political science methodology^10^ to disciplines such as philosophy, political communication and rhetoric. The latter is particularly relevant to elections considering that language use and rhetorical structures are the primary tools for candidates to construct meanings in the world, convey their vision and ideology^11^, and appeal to voters^12,13^.

These disciplines, which are the primary target audience for the dataset presented here, have often focused on State of the Union^14^ and Inaugural addresses as a rich source of information about the president over their time in-office. However, they only reveal part of the speaker’s rhetorical elements that may serve different goals compared to speeches during the elections race (retain approval rates rather than persuade to be voted for). A more informed perspective on the rhetorical traits and appeal strategy of a candidate is achieved by looking at their campaign speeches during the full election cycle. Examples that also use various theoretical frameworks of discourse include Critical Discourse Analysis^15^, or investigating the usage of words that aim to unite and engage with the public (e.g. ‘we’) by the speaker^16,17^.

In the context of US presidential elections, previous work^18^ studied a small number of campaign speeches (30) of candidates Barack Obama and Mitt Romney during the 2012 elections cycle, extracted from the American Presidency Project^19^. The study focused on the rhetorical mechanisms that each candidate utilized and the extent to which they were successful in approaching the voters. The understanding of such mechanisms is critical, particularly when one considers that, recently, election cycles globally, occur in highly polarized political environments, among high levels of citizen distrust to political actors. The importance of such studies became evident during the 2016 election cycle, where a number of studies analyzed the rhetoric of Donald Trump and Hillary Clinton: studies on candidate’s verbal and non-verbal elements that aim to affect the voters^20^, the impact on particular social groups and what that meant for the dynamics of social change^21^, what elements characterize a charismatic speaker or leader^22,23^, and how these are affected by the campaign stage at the time of the speech^24^ or ongoing (inter-)national crises^25^.

The aforementioned studies on US presidents and presidential candidates, have developed datasets for their studies that have a relatively small sample size or limited scope, or exhibit less source variation, as suited to the addressed tasks (The American Presidency Project or The Miller Center^26^, or The Annenberg/Pew Archive of Presidential Campaign Discourse^27^). Furthermore, they may contain different speech types (interviews, rallies, debates, TV spots^28,29^), thus containing inconsistent rhetorical structures, or may include only limited details on the data curation, even when the text is extracted by automated captioning systems^30^, thus prone to large error rates if not well-curated. The presented dataset is, to our knowledge, the first organized attempt at providing a corpus of campaign speeches of the main presidential and also, vice-presidential, candidates, who are often understudied. The authoritative data sources that were utilized, the strict criteria of creation and transparent curation process, ensure the veracity required by rigorous statistical analyses and the high stakes of studies on elections and speaker’s rhetorical strategies^31,32^.

## Methods

### Data timespan and sources

The dataset (data hosted on Zenodo^33^) contains speeches delivered by the Republican (Donald Trump, Mike Pence) and Democratic (Joe Biden, Kamala Harris) ticket candidates for the presidential and vice-presidential roles during the period of January 2019 - January 2021. This period covers the time between the official launch of Kamala Harris’ campaign (1/2019) and the Inauguration of Joe Biden as newly elected President (1/2021). For this period, text data from candidates’ campaign speeches were collected from the following sources:

- the Miller Center of the University of Virginia^26^ (https://millercenter.org/);
- Vote Smart^34^ (https://justfacts.votesmart.org/), a non-profit, non-partisan research organization for the collection of information about candidates for public office in the US;
- the Cable-Satellite Public Affairs Network^35^ (C-SPAN, https://www.c-span.org/), which maintains an archive of televised public campaign speeches;
- for the speeches and statements of the Democrats’ ticket for the 2020 elections, data were also collected from their personal Medium blogs^36,37^ (https://kamalaharris.medium.com/, https://medium.com/@JoeBiden).

The collection method employed was web scraping via purpose-built software in Python which is provided in the repository accompanying the present data descriptor. The use of four different data sources was dictated by the need to gather as much data as possible, while abiding by the dataset criteria which we present in the following section. Note that although there was some amount of overlap among the sources, they crucially complemented each other, particularly in terms of candidates. The Miller Center^26^ collects speeches of elected US Presidents, therefore, we could only extract data for Presidents Joe Biden (for January 2021) and Donald Trump, while inclusion of a speech in the collection is an editorial decision by staff of the Miller Center. C-SPAN^35^ focuses on television broadcast speeches, hence we had to complement it with Vote Smart^34^ data to include written statements of the candidates. It should also be noted that C-SPAN and Vote Smart were the main sources for vice-presidential candidate Mike Pence, whose data were the most difficult to gather online. Similarly, for Kamala Harris, her Medium blogging was an important source of data.

### Criteria for data inclusion in the dataset

The modalities and style of speeches delivered during a campaign vary and may comprise oral speeches (e.g. debates or Convention speeches), and written statements (e.g. news articles, press releases). To construct a dataset that is coherent in terms of rhetorical structure in the speeches, irrespective of the delivery medium, certain criteria were specified for a speech to be included in the dataset. In particular:

- the speeches had to be either oral or written, yet partially or fully scripted;
- the speeches should be destined for an audience of voters, i.e citizens who are not politicians or journalists;
- the speeches had to be delivered and predominantly led by the speaker, rather than a co-host or journalist.

Note that all speeches collected from The Miller Center fulfill these criteria; from Vote Smart, those that fall under the types ‘Speeches’, ‘News article’, ‘Statement’, ‘Op-Ed’ were collected, and further filtered to remove those that did not fulfill one or more of the criteria (especially some in the ‘Statement’ group). Regarding C-SPAN, the majority of the speeches were maintained, yet a significant amount of curation was required due to the transcription format (uncurated closed captioning system), and few of the speeches (<1%) were rejected due to low quality of the transcription. All the curation (cleaning and preprocessing) steps are detailed in the following subsections. Furthermore, given these constraints, discussions or interviews during some campaign events, or joint statements with other political figures (e.g. Senators) were not included in the dataset corpus. In some cases, during a candidate’s speech there were interruptions from the audience or interventions from hosts when asked by the speaker. The conversational and debate-like quality of these passages has led us to remove these interactions from our dataset, retaining only the candidate’s segment.

We remark that the campaign speeches of Donald Trump, where he often invited governors or members of his staff or even of the audience on stage for a short intervention, were eligible for our dataset as he remained the main figure leading the event; these interventions were removed at data duration, preserving the speaker’s parts. Secondly, some of Kamala Harris’ speeches had been published on the Senate website or her Medium page in a journalistic tone. A neutral narrative structure quotes directly Harris, as reported speech (e.g. “Harris commented: ‘…’ ”). We have kept these instances, on the ground that they have been reported by a government organization with a factual tone. Reports concerning public letters, were removed from the dataset, as they did not meet the criterion of being a direct address to American citizens.

### Data collection

The data from The Miller Center^26^, Vote Smart^34^ and Medium blogs^36,37^ were collected via web-scraping methods, with code that was developed specifically for the structure of each website. In particular, although The Miller Center provides an API access to its dataset, a dedicated scraper was collected to extract more information per speech (metadata) than what is available through the API. Finally, C-SPAN^35^ allows downloading the results of a search in its records in a spreadsheet that contains the link to each speech. The candidate relevant speeches were identified by searching C-SPAN based on the speaker, the organization publishing the speech, the content series, the event type and a tag - Table 1 presents the search parameters. Following the first step of collecting the URLs of the relevant speeches, each speech was collected via web-scraping methods from the transcript page of each speech. The web-scraping code developed for the data collection is provided in the accompanying GitHub repository, given in the ‘Code availability’ section.

**Table 1:** Parameters used to search on the C-SPAN website for speeches of interest to the present study.

Dates	Person	Organization	Series	Event types	Tag
1/1/2019-31/1/2021	Donald J. Trump	White House, Trump Presidential Campaign	Campaign 2020, Road to the White House 2020	Speeches, Rally	Presidential nomination, campaigns and elections
1/1/2019-31/1/2021	Joe Biden	Biden Presidential Campaign	Campaign 2020, Road to the White House 2020, Historic Campaign Speeches	Speeches	Presidential nomination, campaigns and elections
1/1/2019-31/1/2021	Kamala D. Harris	Harris Presidential Campaign	Campaign 2020, Road to the White House 2020	Speeches, Rally	Presidential nomination, campaigns and elections
1/1/2019-31/1/2021	Mike Pence	White House, Office of the Vice President, Trump Presidential Campaign	Campaign 2020, Road to the White House 2020	Speeches, Rally, Appearance	Presidential nomination, campaigns and elections

### Cleaning and preprocessing

Due to the aforementioned strict dataset requirements and to ensure the veracity of the dataset^38^, the collected text data were cleaned and preprocessed in an iterative way, considering the specificity of each studied speaker and their role.

First, any speeches (henceforth ‘speeches’ will collectively refer to a dataset item, be it a Speech or a Statement or an Op-ed or a News article) that were given during an official visit outside the US, referring to a specific event abroad, or given at press conferences and briefings were removed, as the speaker addresses the local population or journalists rather the American people/voters. These included, for example: speeches commenting on Belarus or the conflict in Nagorno-Karabakh, or the passing of international political figures; joint remarks during official visits, given together with the host and predominantly addressing the local citizens (e.g. ‘Remarks by Vice President Pence at a Breakfast with Prime Minister Varadkar of Ireland’); joint statements with senators or other politicians; proclamations or remarks during signing of official Executive Orders.

Note that the type and occasion of the speeches was dictated by the role of each studied figure. In particular, Donald Trump’s speeches are particularly interactive, as he often addresses the audience or bystanders, poses rhetorical or confirmatory questions, and responds to audience’s reactions (cheering/booing, comments spoken out loud by audience members). These instances of interruption of the speech by audience remarks were removed, yet, the candidate’s speech was retained as he always remains the dominant figure in the campaign event. On the other hand, Mike Pence mostly spoke with the opportunity of invitations at events, receptions, union gatherings or international visits as the representative of the Administration. Hence, many of the collected speeches attributed to him were removed as they did not meet the criteria of the dataset, except for those events that were open to the public. Furthermore, Kamala Harris, as expected in her role of Senator, often released short statements in response to governmental actions or legislative initiatives or sent official letters to the President. Note that many of them were co-signed by fellow Senators, with whom she often issued co-statements. In these cases, only Harris’ direct quotes in the statement were maintained, and any letters and joint statements with multiple Senators were removed. Finally, we remark that Joe Biden’s speeches were the most compatible to a candidate’s speech addressed to the public in terms of speech structure; only a few that were addressed to an audience abroad were removed. Details on the filters and code that applied them are included in the Data Records section.

Particularly challenging was the curation of the text data collected from C-SPAN^35^, as the transcripts are obtained via closed captioning and had not undergone any curation. Closed-captioning systems consist of Automatic Speech Recognition (ASR) statistical models that map audible speech to its text equivalent (transcription). Although ASR technology is very advanced, with error rates as low as 7% for English, the performance of such systems varies significantly depending on background noise, acoustic conditions, speaker clarity, dialect, environmental conditions^39^. Importantly, in outdoor environments - as very often was the case for the speeches of interest to this work, given that most rallies were held outdoors - wind interference, background ambient noise, or speaker distance from microphone may significantly degrade the performance of the closed captioning system. In addition, the ASR performance degrades even more when closed-captioning occurs in a streaming fashion (again the case for C-SPAN), rather than a one-off transcription of the complete audio sample.

The cleaning of the C-SPAN data began, first, by removing speeches that were not compatible with the determined criteria. Second, the start and end points of the candidate’s speech were manually identified, and only the transcript in between was retained whenever it corresponded to speech of one of the candidates. This removed any introductions to the speaker, delivered by the event host, any interventions from invited speakers on stage or the audience, or even any lyrics from the music score played at each speaker’s campaign event (often also captured by the closed captioning system).

To ensure data quality, a set of cleaning steps (Algorithm 1) were conducted immediately after each transcript was extracted from the source website, resulting in the ‘raw text’ in the dataset. Next, non-compatible speeches or non-compatible segments of speeches were removed, a process conducted in part manually, carefully examining the speeches, followed by a set of preprocessing steps (Algorithm 2). The code that implemented these cleaning and preprocessing steps is provided in the accompanying GitHub repository, given in the ‘Code availability’ section and described in the ‘Data Records’ section.

#### Algorithm 1

Cleaning of speech transcript.

#### Algorithm 2

Pre-processing of speech transcript.

## Data Records

### Overview

The dataset is publicly available on Zenodo^33^ (10.5281/zenodo.14785782) and GitHub (https://github.com/ichalkiad/datadescriptor_uselections2020). It is comprised of two parts: the raw data which were the output of the web-scraping process (located in the ‘data’ folder), and the pre-processed data (located in the folder ‘data_clean’) which contain the dataset after the cleaning and pre-processing steps. A summary of the curated dataset is presented in Table 2. Table 3 details the attributes per data item in the collected dataset, in compliance with FAIR^40^ principles.

**Table 2:** Number of speeches that remained in the dataset after the curation process, per source and per candidate.

Candidate	Speech source				
(alphabetically listed based on surname)	The Miller Center	VoteSmart	C-SPAN	Medium	Total
**Joe Biden**	1	176	106	136	419
**Kamala Harris**	—	89	36	39	164
**Mike Pence**	—	92	44	—	136
**Donald Trump**	29	211	97	—	337

**Table 3:** Collected dataset attributes.

Attribute	Type	Content			
		The Miller Center	Vote Smart	C-SPAN	Medium
**SpeechID**	string	Unique speech identifier in the form: source initials∣president’s initials∣day∣month∣year of speech∣increasing speech count E.g. MCJB201202120: Joe Biden 20/01/2021, 20th collected speech from The Miller Center			
**POTUS**	string	President’s name			
**Date**	string	Date of speech delivery			
**SpeechTitle**	string	Title of the speech			
**Type**	string	—	Statement, or Speech, or News Article, or Op-Ed	Speech, Rally, Appearance	—
**RawText**	string	Speech transcription as scraped from the website			
CleanText^*^	string	Speech transcription after cleaning and preprocessing			
**SpeechURL**	string	Speech URL			
**Summary**	string	Short overview of the speech	Set of topic labels for the speech (provided by C-SPAN)	Short overview of the speech	Short overview of the speech (when available)
**Source**	string	Institution of speech archive (e.g. The White House, Trump/Pence campaign)	—	C-SPAN speech ID	—
**Original Source**	string	—	URL of initial speech host (e.g. whitehouse.gov, govinfo.gov)	—	—
**Location**	string	—	Location where speech was given	—	—

Dashes (—) correspond to “null" values in the data structure.

^*^ The field ‘CleanText’ is present in cleaned dataset only, i.e. after preprocessing.

### Details on the dataset and code repositories

The raw and clean data folders are organized in subfolders, one for each speech source and speaker. The raw data contain the text of the speeches after the initial cleaning (‘rawtext_{speaker name}’) and potentially a list of identifiers for speeches that were removed as non-compliant with the dataset criteria (‘drop_speech_id.tsv’). In the case of C-SPAN^35^, where significant manual curation was needed, the results of the website search are included (‘cspan_{speaker name}.csv’) as well as intermediate files (‘rawtext_droptitles_{speaker name}’, ‘rawtext_droptitles_{speaker name}_edit2’, automatically created following the accompanying curation code, ‘rawtext_droptitles_{speaker name}_edit1’, manually created and used by the accompanying curation code), as well as a folder (‘specialcleanneeded’) with the transcripts of some particularly long speeches stored individually. The latter was required only for the data curation; these speeches are integrated into the dataset folder (‘data’).

In terms of data organization, the dataset is divided on two levels: first, per source, and second, per candidate. We do not provide a single data structure that contains documents from all four sources and speakers. This is to allow for more efficiency and flexibility in how the data are stored and used. In terms of construction criteria and metadata fields (that may contain ‘null’ if certain information is not present, please refer to Table 3), all dataset parts are consistent with each other.

Both the raw and the curated data are provided in three formats to facilitate different processing pipelines:

- tab-separated file (^*^.tsv) for easy data inspection and format familiarity,
- JSONL (^*^.jsonl), i.e. JSON format, one record per file line, for accessibility and easy integration in case of streaming data processing pipelines,
- Apache Parquet^41^ format (^*^.parquet), which preserves data types and is very useful for Big Data setups and workflows.

All provided formats are particularly easy to handle and are readily handled by popular data wrangling software libraries such as Python’s pandas. The accompanying code repository contains data collection and curation code, which are presented next, and code for the technical validation exercise and use case which is presented in Section ‘Usage Notes’. The data collection and curation code is included in the webcollect/ and src/ folders of the GitHub repository, while supporting topic dictionaries are provided in the folder lexica/.

The ‘webcollect’ folder contains the scraping scripts per speech source (site structure at the time of the data collection):

- *webscrape_bidenmedium.py* and *webscrape_harrismedium.py*: each contains the scraping code for the Medium blog of candidates Joe Biden and Kamala Harris. The latter script contains the function that extracts the speech transcript (*get_president_speech*), which is common in both scripts.
- *webscrape_cspan.py*: the script loads the spreadsheet extracted from C-SPAN, containing the URLs to potentially relevant dataset speeches. It then iterates over each item and extracts the speech using its transcription URL.
- *webscrape_miller_daterange.py*: the script extracts the list of speeches per president, and scrapes the transcript of each speech that falls in the focus period.
- *webscrape_votesmart.py*: the scripts iterates over the relevant speech types per candidate, obtains the URL of each speech, and subsequently extracts the transcript.

The ‘src’ folder contains the scripts for the data curation. In particular:

- *quotes.py*: auxiliary script containing the Unicode code points for quotation marks. The script is used to ensure that all quotes have a uniform Unicode representation to facilitate their processing.
- *utils.py*: contains all pre-processing code, namely the designed regular expressions, functions that operate on the raw text and pre-process it, as well as wrapper cleaning functions that consider the specificities of each source, apply the corresponding pre-processing steps and store the cleaned data.
- *datacurate*_{*speaker surname*}*.py*: one curation script per candidate, calling the cleaning function that corresponds to each source.

The ‘lexica’ folder contains supporting word dictionaries, extracted from the Oxford Dictionaries series for further processing of the text data when needed^42–44^ - an example usage is presented in sections ‘Technical Validation’ and ‘Usage Notes’. An auxiliary file (tstats_target_8.pkl^45^) utilized when validating the dataset is also included.

## Technical Validation

For the technical validation of the dataset, we remark that it is inherently difficult to define ‘high-quality’ text data^38,46^, especially when the dataset is intentionally provided with minimal processing so that it is usable in a variety of applications. These may include dictionary methods that primarily consider only which words are present in the data, grammatical or syntactical analysis applications that require full textual information including punctuation, or word-embedding/Large Language Models methods that may also internally operate with ‘sub-word’ units, hence overlooking textual data quality and focusing on quantity. The construction of the election speeches dataset presented in the manuscript, considers two primary factors as affecting the veracity of the data: data provenance and removal of textual noise from the data before further processing. The latter includes aspects such as the type of encoding of text data or artifacts that may have been introduced at the data collection stage (e.g. if done via optical character recognition, web-scraping).

Regarding provenance, the veracity of the presented elections dataset is first guaranteed by the chosen sources: an academic center focused on research on the presidency (The Miller Center^26^); official blog accounts of the candidates, run by their campaign teams; C-SPAN^35^, a nonprofit public service operating for over 45 years; and Vote Smart^34^, a nonprofit, non-partisan research organization, set-up by former US presidents and supported by politicians across the political spectrum, which has been awarded for transparency and quality of service, providing factual information about people running for public office. Furthermore, the included dictionaries are constructed based on authoritative sources (Oxford Dictionaries), which ensure that they are well-maintained and carefully curated.

**Fig. 1:**
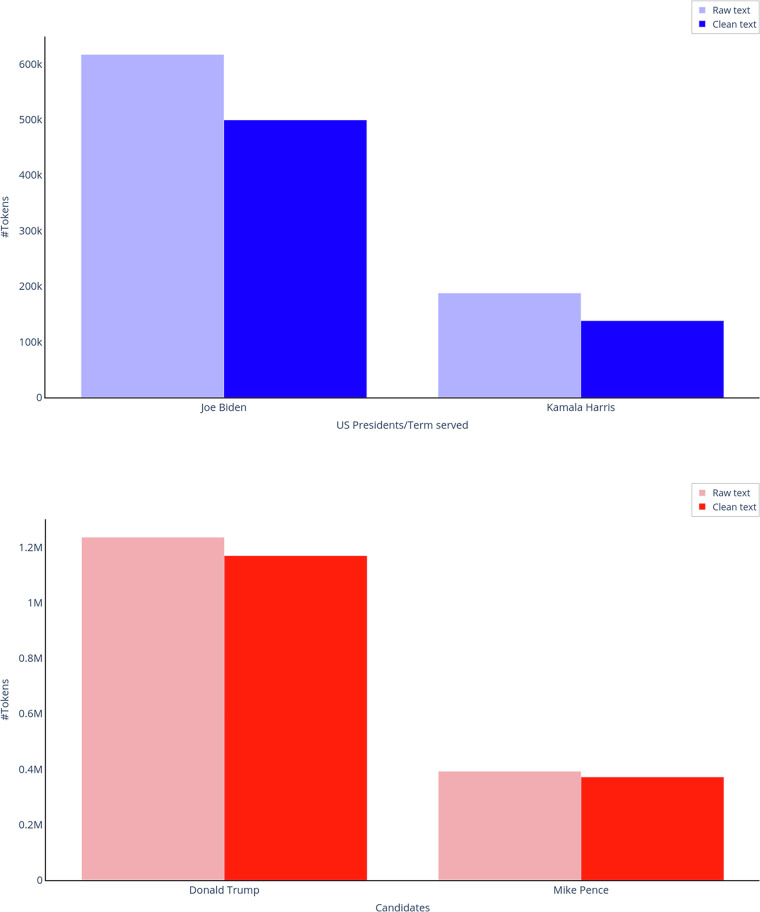
Raw and post-curation number of tokens (text units after splitting on whitespace) per speaker.

Regarding the aspect of noise in the text, data quality is ensured by the criteria of building the dataset, where we require speeches that, to a large extent, have been scripted and curated by the speaker’s team, and also by the detailed cleaning and pre-processing steps. For the latter, the number of tokens (elements separated on whitespace, no words/articles removed) per speaker before and after the curation process are presented in Fig. 1. We remark the high percentage of retained tokens for all speakers (median of  ~ 87%), which signifies the quality of the curation, as minimal amount of information was removed.

**Fig. 2:**
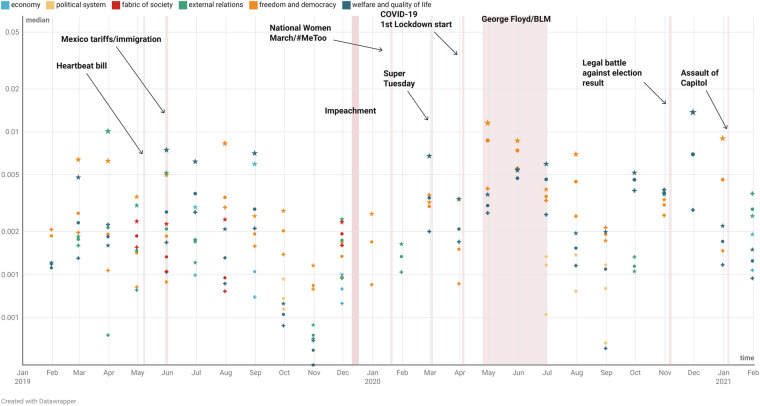
The chart shows the proportion (logarithmic median over campaign speeches, determines the bubble size) of the dominant topics that US (vice-) presidential candidates covered in their public speeches in the period leading to the 2020 elections up until January 2021. The time covariate in the structural topic model denotes a countdown to January 31, 2021. Democrats are denoted with *, Republicans with  + and  ⋅ (dot) is the topic proportion in the campaign speeches overall.

**Fig. 3:**
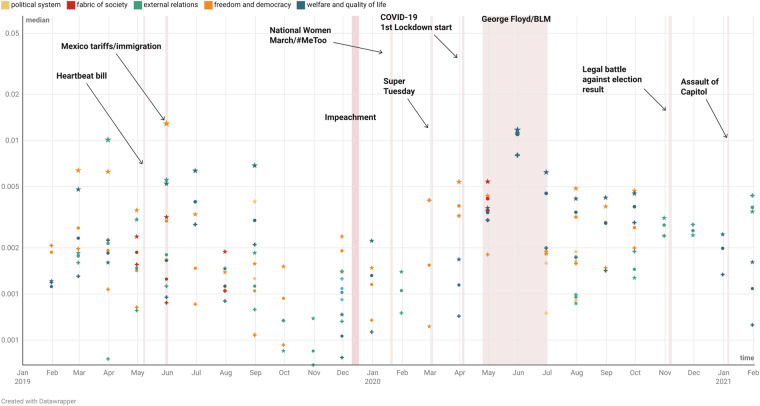
The chart shows the proportion (logarithmic median over campaign speeches, determines the bubble size) of the dominant topics that US (vice-) presidential candidates covered in their public speeches in the period leading to the 2020 elections up until January 2021. The time covariate is a countdown until the first significant event (Heartbeat bill, May 7, 2019) and a step function changing level at each one of the remaining events. Democrats are denoted with *, Republicans with  + and  ⋅ (dot) is the topic proportion in the campaign speeches overall.

Finally, the quality of the dataset is verified by utilizing it in a machine learning topic modeling application, in such a way that high-quality data are necessary to obtain meaningful outcomes. This is because we do not just discover topics that are summarized as collections of words, but instead map each discovered ‘topic’ to reference topics in political science^45,47^, using an algorithm that requires a significant overlap between the text data and the politics-related Oxford Dictionaries word lists that we constructed (Table 4). Specifically, we explicitly use these word lists in two stages in our work to ensure data veracity: first, when pre-processing the text that is fed into the topic model to use document words that are present in the collected Oxford Dictionaries, as opposed to using all words in the corpus, perhaps with some occurrence thresholds as is common practice in topic modeling. Second, in the topic labelling procedure of Algorithm, where the Oxford University authoritative dictionaries are used as a basis onto which we project both the topics that the topic model estimates, and the reference topics that we want to use to label the estimated topics. This application, presented in detail in the Supplementary Information as an example use case, illustrates that the presented dataset contains statistical structure that allows one to conduct automated text data analyses with meaningful, interpretable outcomes. Illustrations of such analyses are Figs. 2 and 3, which present the proportion (in a logarithmic scale y-axis) of each Manifesto project topic^47–49^ in the dataset, over the period of focus. The proportions per party (Democrats are denoted with *, Republicans with +) and over the complete dataset (denoted with  ⋅ ) are plotted. Figure 2 is constructed based on topics obtained from an STM with a time covariate that denotes a countdown to January 31, 2021. Figure 3 is constructed based on topics obtained from an STM with a time covariate that is a step function with different levels to denote significant events throughout the studied period. We do indeed observe meaningful topics being more present in the election debate in relevant periods. For instance, in Fig. 2 we observe that in June 2019, for both parties the *economy* was the prevalent issue, followed by *external relations, freedom and democracy* for the Democratic party, and *external relations, fabric of society* for the Republican party. This is consistent with the fact that at the time imposing tariffs on Mexico was discussed as a way to stop incoming immigration to the US. The fact that the analysis of the data agrees with the observed political context at the time indicates that the dataset indeed contains statistical structure that is descriptive of the politics of the period.

**Table 4:** Details of the sources we used to build the dictionary. Dictionary size is measured in number of words.

Dictionary	Size	Source
US Politics	1882	https://www.senate.gov/about/glossary.htm https://www.vocabulary.com/lists/izmfh6e4/presidential-parlance https://www.oxfordreference.com/display/10.1093/acref/9780195176612.001.0001/acref-9780195176612
Political Science	11360	https://www.oxfordreference.com/display/10.1093/acref/9780199670840.001.0001/acref-9780199670840 https://www.oxfordreference.com/display/10.1093/acref/9780191890949.001.0001/acref-9780191890949 https://www.oxfordreference.com/display/10.1093/acref/9780198759430.001.0001/acref-9780198759430 https://www.oxfordreference.com/display/10.1093/acref/9780191834837.001.0001/acref-9780191834837 https://www.oxfordreference.com/display/10.1093/acref/9780199683581.001.0001/acref-9780199683581 https://www.boundless.com/immigration-resources/immigration-glossary/ https://www.uscis.gov/tools/glossary
Politics and Government	2,450	https://www.excellentesl4u.com/esl-politics-vocabulary.html
		https://www.scholastic.com/teachers/articles/teaching-content/vocabulary-political-words/
		https://www.macmillandictionary.com/thesaurus-category/british/general-words-relating-to-politics-and-government
		https://www.vocabulary.com/lists/183710
		https://myvocabulary.com/word-list/politics-vocabulary/
		https://www.collinsdictionary.com/word-lists/government-types-of-government
Epidemics	1,887	https://www.oxfordreference.com/view/10.1093/acref/9780195314496.001.0001/acref-9780195314496
Media	7,360	https://www.oxfordreference.com/view/10.1093/acref/9780191826740.001.0001/acref-9780191826740 https://www.oxfordreference.com/view/10.1093/acref/9780191803093.001.0001/acref-9780191803093 https://www.oxfordreference.com/view/10.1093/acref/9780191863592.001.0001/acref-9780191863592 https://www.oxfordreference.com/view/10.1093/acref/9780191800986.001.0001/acref-9780191800986 https://www.oxfordreference.com/view/10.1093/acref/9780199646241.001.0001/acref-9780199646241 https://www.oxfordreference.com/view/10.1093/acref/9780199587261.001.0001/acref-9780199587261 https://www.ket.org/education/resources/drama-glossary/#word-wall-printouts https://myvocabulary.com/word-list/media-literacy-vocabulary
Religion	1,433	https://www.oxfordreference.com/view/10.1093/acref/9780191823527.001.0001/acref-9780191823527 https://www.oxfordreference.com/view/10.1093/acref/9780191816819.001.0001/acref-9780191816819 http://phrontistery.info/church.html https://hfgb.org/becoming-catholic/catholic-vocabulary/ https://pages.ucsd.edu/~dkjordan/xy/xydocs/CatholicTerms.html https://referenceworks.brillonline.com/browse/vocabulary-for-the-study-of-religion https://www.dailywritingtips.com/30-religious-terms-you-should-know/ https://www.macmillandictionary.com/thesaurus-category/british/words-used-to-describe-religious-people http://www.manythings.org/vocabulary/lists/c/words.php?f=christmas_religious https://www.collinsdictionary.com/word-lists/religion-religions
Baseline dictionary	245,318	Academic wordlist: https://www.wgtn.ac.nz/lals/resources/academicwordlist
		Oxford 5000 word list: https://www.oxfordlearnersdictionaries.com/wordlists/oxford3000-5000
		Oxford World-Place names: https://www.oxfordreference.com/view/10.1093/acref/9780199580897.001.0001/acref-9780199580897
		https://www.rollingstone.com/music/music-lists/100-greatest-artists-147446/
		https://www.biographyonline.net/people/100-most-influential.html
		https://time.com/collection/most-influential-people-2018/
		https://time.com/collection/100-most-influential-people-2019
		Unix built-in default dictionary

## Usage Notes

Our dataset is released under a CC BY-NC 4.0 International license (Creative Commons Attribution-Non Commercial 4.0, https://creativecommons.org/licenses/by-nc/4.0/deed.en). Users are welcome to share and adapt the dataset as long as they provide appropriate credit, a link to the license, and indicate if changes were made. Note that to be fully compliant with our sources, the dataset may not be used for commercial purposes.

The formats that the dataset is distributed in (tsv, jsonl, parquet) and the FAIR principles for its construction ensure that it is readily and easily reusable. Please see below for examples in Python on how to read in the dataset:

Furthermore, considering the data collection criteria and databases, this text dataset of campaign speeches significantly informs research in fields such as political science, rhetoric, media and communication studies, and political marketing. The rich collection of campaign speeches, including vice-presidential candidate speeches which is uncommon in general, while carefully curated to have consistent rhetorical structure, enables testing several hypotheses and research questions. These may pertain both to inter- and intra-party studies, such as: whether the Vice-President’s speeches contribute more or less significantly to the Party’s campaign ticket; what is the connection between campaign timelines and the rhetorical adjustments, or lack thereof, to specific events during the full length of the campaign; how candidates’ rhetoric fuels or diminishes polarization between party supporters and its evolution over time and events. Importantly, the dataset can be also used in studies of temporal nature, such as detecting speech change points, which may include change of thematic focus of the candidates, emergence of polarized speech etc.

## Supplementary information

Supplementary Information

## Data Availability

The code for the collection, curation and topic modeling case study is available on GitHub (https://github.com/ichalkiad/datadescriptor_uselections2020) under the MIT License. The software was developed in Python v3.8 and the following packages (and versions) were used: numpy (1.23.5), scipy (1.9.3), pandas (1.5.2), flashtext (2.7), nltk (3.8), plotly (5.11.0), scikit-learn (1.2.1), pyarrow (11.0.0). The structural topic model was run in R (4.4) using the stm (1.3.7) package.
