# Supplementary material for: A text dataset of campaign speeches of the main tickets in the 2020 US presidential election
Source: Sci Data. 2025 Apr 19;12:662. doi: 10.1038/s41597-025-04681-x (PMC12009287; doi:10.1038/s41597-025-04681-x)
Supplement: Supplementary file 1 — Supplementary Information [file 41597_2025_4681_MOESM1_ESM.pdf]

A text dataset of campaign speeches of the main tickets in the 2020 US presidential election: Supplementary information

Ioannis Chalkiadakis[1], **Louise Anglès d'Auriac**[2], **Gareth W. Peters**[3], **and Divina Frau-Meigs**[4]

[1]Institut des Systèmes Complexes de Paris Île-de-France, CNRS, Paris, 75013, France
[2]ED 625 MAGIIE, Université Sorbonne Nouvelle, Paris, 75012, France
[3]Department of Statistics and Applied Probability, University of California Santa Barbara, 93106, USA
[4]ED 625 MAGIIE, Université Sorbonne Nouvelle, Paris, 75012, France
[*]corresponding author: Ioannis Chalkiadakis (ioannis.chalkiadakis@cnrs.fr)

ABSTRACT

Supplementary information to the main manuscript, which include a case study that illustrates the statistical structure present in the data. Furthermore, the supplementary material includes: i) a step-by-step procedure to run the code that reproduces the case study; ii) an algorithm for the automatic labeling of topics discovered by a topic modeling procedure, given reference dictionaries and topics, to accompany the software implementation of the code repository (https://github.com/ichalkiad/datadescriptor_uselections2020).

Example usage reproducing the structural topic modeling study

In this section, the pipeline for conducting a structural topic modeling (STM[1]) exercise will be presented, showing how the clean data provided are post-processed before being fed in the STM algorithm. The flow chart in Figure 4 provides an overview of the procedure for collecting, curating the data and running the present case study.

For the topic modeling study of the agenda of the race, any punctuation and numbers were removed, as well as common stopwords together with a list of additional stopwords containing e.g. the names of the candidates or words such as 'hello', 'thank you' etc, often spoken in campaign speeches. Finally, all tokens were stemmed using the Snowball stemmer of NLTK[2].

Structural topic modeling to identify the agenda of the campaign

In this section we detail the procedure that was followed to estimate and evaluate the probabilistic structural topic model for the automated thematic segmentation of the 2020 elections race. Like the typical Latent Dirichlet Allocation (LDA) topic modeling approach[3], the STM assumes that each word in a document comes from one single topic, and each document is represented by the proportion of its words coming from each one of K topics. However, the main benefit of using the STM versus relying on the baseline LDA is that the former allows to condition the topic and vocabulary selection per document on a set of external covariates. In our case, the introduced covariates are the candidate's party (Democrats or Republicans) and a time covariate, for which we experimented with two different choices: either a covariate that is a piecewise constant function with different levels to indicate significant events in the race, which are identified in the list below, or a covariate that is a countdown to the end of the studied period (January 31, 2021):

- Heartbeat Bill: May 7, 2019
- Tariffs on goods from Mexico, in connection with the immigration discussion being in the spotlight: May 30, 2019
- Donald Trump officially accused of abuse of power and obstruction of Congress: December 10, 2019
- First National Women March/MeToo movement: January 19, 2020
- Super Tuesday, beginning of Joe Biden's winning streak: March 3, 2020
- 1st COVID-19 lockdown: April 5, 2020
- Beginning of a series of "Black Lives Matter" movement demonstrations: May 1, 2020

- Official announcement of Kamala Harris as running mate in Joe Biden's ticket: August 11, 2020

- Joe Biden declared winner of the elections, Donald Trump starts a legal battle to overturn the election result: November 7, 2020

- Nomination of Joe Biden as POTUS, assault of Capitol: January 6, 2021

To begin with, we look for the topic evolution per month between January 2019 and January 2021. To discover a smoother topic variation and avoid spurious topic detection due to insufficient data, for each month we consider a 3-month backward-looking window (current and preceding two months) that shifts with a monthly step. Note that in January 2019 and February 2019 we only used the available data and did not extend before January 2019.

Furthermore, instead of following the usual practice of building a lexicon based only on tokens present in the text corpus after some adjustments to account for stopwords and low-frequency words, we instead utilized the lexicon of Table **??** and its intersection with the text corpus of each 3-month window. The lexicon $\mathbb{D}$ is comprised of $J = 7$ sub-dictionaries on the following topics: US Politics, Political Science, Politics and Government, Epidemics, Media, Religion, and a baseline English dictionary. Constraining the topic model in this way, allows us to detect more interpretable topics and benefit from improved computational efficiency due to reduced vocabulary size in the STM.

Regarding the specifics of applying the STM, we applied the sparse additive generative model (SAGE[4]) which considers topics to be sparse deviations from a corpus wide baseline of token selection. Intuitively, SAGE topic models model the difference in token log-frequency from a baseline distribution, facilitating regularization of important term selection. Hence, the output topic model is more robust to limited training data, and the topics are more interpretable as words are attributed to topics only when they have enough occurrences to exceed their baseline prior.

For each 3-month window we fit topic models with $K \in \{10, 15, 20, 25\}$ topics. Each model with a given number of topics is estimated with an Expectation-Maximization[5] algorithm using multiple restart points. Among the highest likelihood models, the one whose topics have the highest semantic coherence is selected, provided it is sparse enough. The metric of semantic coherence is maximized when the most likely words in a given topic frequently co-occur together, which is correlated with human judgment of topic quality[6]. Specifically, let $D(v, v')$ denote the number of times words v, v' co-occur together. For a list of the P most probable words in topic k, the semantic coherence for topic k is measured as follows:

$$C_k = \sum_{p=2}^{P} \sum_{j=1}^{p-1} \log \left(\frac{D(v_p, v_j) + 1}{D(v_j)} \right). \tag{1}$$

Sparsity is measured in terms of the model parameters pertinent to the words, which characterize the content covariate and the interaction between the topic and covariate groupings. Among the selected models for $K \in \{10, 15, 20, 25\}$, we investigate which achieved the best fit: first, the matrix containing the log probabilities of seeing word w conditional on topic k is checked for problematic tokens, i.e. tokens that exclusively associate with a particular topic, and second, an additional test[7] is applied to acquire a rough assessment of the number of selected topics. Among those fits that pass the two tests, we select the model fit that achieved the highest evidence lower bound during estimation. Note that the selected number of topics per 3-month period may vary.

Identifying meaningful topics and topic label attribution

To reduce the number of identified topics, hence assist with their interpretation, a LASSO-based regression is performed to detect positive correlations among them[8,9]. When such correlations are detected, the correlated topics are treated as a single topic. At this stage it should be noted that standard, unsupervised topic modeling approaches[10–12] output collections of words that are deemed to represent topics; however, it is up to the researcher to determine at a later stage what semantic theme each word collection captures. Often, this is challenging to achieve, hence it has been a point of critique against topic modeling. Inspired from previous work[13,14], Algorithm 3 presents an automatic procedure to label word collections obtained from a topic model, using a set of dictionaries that each is known to describe a particular theme. We refer to these dictionaries as 'annotation dictionaries' and denote them $\mathbb{D}^\alpha = \{\mathbb{D}^\alpha_1, \ldots, \mathbb{D}^\alpha_I\}$. These dictionaries may have been associated with their theme by some machine learning procedure or they may have been annotated by an expert. In the present case study, we utilized publicly available data (word collections) of a previous work[15], where the authors have associated words from political documents with topics that have been established to be prevalent in political science documents over the long-time research effort that constitutes the Manifesto project[16–18]. These topics are the following: *external relations, freedom and democracy, political system, economy, welfare and quality of life, fabric of society, social groups*, which we consider as 'gold' label topics, and onto which we map the word collections (namely the topics) estimated from the structural topic model that we developed. Note that

the Manifesto project constitutes a reference for political party policy documents, is frequently updated and curated by experts, and, importantly, includes US political party documents of the 2020 elections; hence, the aforementioned topics are relevant to our study and corpus. The Algorithm proceeds as follows:

- Step 1: Each cluster set c contains a subset of topics estimated from the STM, which have been determined correlated by the LASSO procedure[8,9]. The proportion of each topic in c per document in M is extracted, and then the median $\hat{m}_c$ over all of these proportions is estimated.

- Step 2: Compute the dominant cluster sets $\tilde{\mathscr{C}}$ as those whose median of Step 1 is in the 6th decile of all cluster set medians.

- Step 3: For each of the dominant topics $\tilde{c} \in \tilde{\mathscr{C}}$, we aim to extract its representative documents, based on which we will assign a thematic label to the word collection that constitutes the topic. We consider as representative those documents whose proportion of at least one of the topics of $\tilde{c} = \{\gamma_{\tilde{c}_1}, \ldots, \gamma_{\tilde{c}_L}\}$ is greater than 80%. If no such documents exist, we consider a single representative document, the one with the highest proportion of any of the topics of $\tilde{c}$. Note that the threshold of 80% was empirically determined to be such as to contain a sufficient number of documents that correspond to the topic theme and facilitate its characterization. A higher threshold would identify more specialized documents, making the topic characterization less general, while a lower threshold would contain too many documents to allow for a specific enough topic characterization. It is, however, a choice that the user can make depending on their application needs.

- Step 4: The words that the STM has associated to each topic that belongs to a dominant cluster set $\tilde{c}$ are combined with words from metadata (e.g. the summaries) of the representative documents for $\tilde{c}$. These words form the set $W_{\tilde{c}}$.

- Step 5: For each $\tilde{c}$, a vector is constructed where each element contains the ratio of the intersection of $\tilde{c}$ with each sub-dictionary $\mathbb{D}^j$, over the total number of words in common. This vector is denoted as $\boldsymbol{p}_{\tilde{c}}^{\mathbb{D}} \in \mathbb{R}^J$ and its elements are computed as follows:

$$p_{\tilde{c},j}^{\mathbb{D}} = \frac{|W_{\tilde{c}} \cap \mathbb{D}^j|}{\sum_{m=1}^{\mathbb{D}} |W_{\tilde{c}} \cap \mathbb{D}^m|}.$$

- Step 6: For each dictionary in the annotation dictionary set $\mathbb{D}^\alpha = \{\mathbb{D}_1^\alpha, \ldots, \mathbb{D}_I^\alpha\}$, a vector is constructed where each element i contains the ratio of the intersection of annotation dictionary $\mathbb{D}_i^\alpha$ with each sub-dictionary $\mathbb{D}^j$, over the total number of words in common. This vector is denoted as $\boldsymbol{a}_i^{\mathbb{D}} \in \mathbb{R}^J$ and its elements are computed as follows:

$$\alpha_{i,j}^{\mathbb{D}} = \frac{|\mathbb{D}_i^\alpha \cap \mathbb{D}^j|}{\sum_{m=1}^{\mathbb{D}} |\mathbb{D}_i^\alpha \cap \mathbb{D}^m|}.$$

- Step 7: In this step we measure the similarity of each vector $\boldsymbol{p}_{\tilde{c}}^{\mathbb{D}}$, characterizing topic $\tilde{c}$, with vectors $\boldsymbol{a}_i^{\mathbb{D}}, i = 1 \ldots, I$, characterizing the similarity of the annotation dictionary with the dictionary we used in the STM. The similarity is quantified as one minus the distance between the two vectors, which is taken to be the cosine distance between the vectors. In so doing we obtain a set of I values that quantify how thematically close topic $\tilde{c}$ is to each of the I annotation dictionaries. In our case, we know that the annotation dictionaries correspond to the Manifesto project topics: *external relations, freedom and democracy, political system, economy, welfare and quality of life, fabric of society, social groups*. We can then order, in a decreasing sequence, the similarity of topic $\tilde{c}$ with each of the annotation dictionaries.

- Step 8: Select as theme for topic $\tilde{c}$ the theme with the highest similarity, namely the first element of the ordered sequence of Step 7.

Example data processing pipeline

The code for the case study is provided in the folder `stm/` in the accompanying code repository. It contains the following files, which are presented in their execution order, as the number in the filename also indicates:

1. *postscrape_elections2020_0.py*: the data per speaker are combined to include all speech sources in a single file and the speeches are split into sentences. The script also plots basic summary statistics.

2. *postprocess_elections2020_1.py*: the dataset is tokenized, using the provided dictionary, and summary statistics after tokenisation are plotted.

3. *prepare_data_stm_elections2020_2.py*: the dataset is split into the time windows where the STM will be applied and the time covariates are constructed. To ensure full control over the text processing, the script also constructs the document-term matrices and vocabulary per time window, which will be loaded in the STM script.

4. *stm_parallel_onepiece_elections2020_3.R*: R script that estimates the STM per time window, runs a number of diagnostic routines (*searchK*, *check_residuals*, *sageLabels*) and computes metrics for model selection.

5. *gatherresults_stm_elections2020_4.R*: summarizes the output of the STM estimation over all time window folders.

6. *postprocess_stm_elections2020_5.py*: the script analyses the best topic model per time window, summarizes the STM-discovered topics and outputs a summary spreadsheet that contains topics information, the corresponding time window, and the median proportion of the topic per party and overall.

7. *postprocess_stm_goldtopics_distributionOndictionaries_elections2020_6.py*: estimates the distribution of the most representative words per reference topic (identified as described in the previous section) over the dictionaries we utilized.

8. *postprocess_stm_distributionOndictionaries_elections2020_7.py*: estimates, for each time window, the distribution of the STM-discovered topics (identified as described in the previous section) over the dictionaries we utilized.

9. *postprocess_stm_goldtopics_match_elections2020_8.py*: using the output of the two previous scripts and Algorithm 3, it labels the STM-discovered topics with one of the reference topics for political science.

10. *postprocess_stm_potustopics_elections2020_9.py*: summarizes STM and topic labeling output into a spreadsheet for easy visualization and analysis.

11. *postprocess_stm_significance_timecovariate_10.py*: assesses the statistical significance of the time covariate by computing the percentage of the coefficients of the spline for the time covariate that were statistically significant at the 5% level.

References

1. Roberts, M. E., Stewart, B. M. & Tingley, D. stm: An R package for Structural Topic Models. *J. Stat. Softw.* **91**, 1–40 (2019).

2. Bird, S., Klein, E. & Loper, E. *Natural Language Processing with Python - Analyzing Text with the Natural Language Toolkit* (O'Reilly Media, Inc., 2009).

3. Blei, D. M., Ng, A. Y. & Jordan, M. I. Latent Dirichlet Allocation. *J. Mach. Learn. Res.* **3**, 993–1022 (2003).

4. Eisenstein, J., Ahmed, A. & Xing, E. P. Sparse Additive Generative Models of Text. In *Proceedings of the 28th International Conference on International Conference on Machine Learning*, ICML'11, 1041–1048 (Omnipress, Madison, WI, USA, 2011).

5. Dempster, A. P., Laird, N. M. & Rubin, D. B. Maximum Likelihood from Incomplete Data via the *EM* Algorithm. *J. royal statistical society: series B (methodological)* **39**, 1–22 (1977).

6. Mimno, David and Wallach, Hanna and Talley, Edmund and Leenders, Miriam and McCallum, Andrew. Optimizing Semantic Coherence in Topic Models. In *Proceedings of the 2011 conference on empirical methods in natural language processing*, 262–272 (2011).

7. Taddy, M. On Estimation and Selection for Topic Models. In *Artificial intelligence and statistics*, 1184–1193 (PMLR, 2012).

8. Meinshausen, N. & Bühlmann, P. High-Dimensional Graphs and Variable Selection with the Lasso. *The Annals Stat.* **34**, 1436–1462 (2006).

9. Zhao, T., Liu, H., Roeder, K., Lafferty, J. & Wasserman, L. The huge package for high-dimensional undirected graph estimation in R. *J. Mach. Learn. Res.* **13**, 1059–1062 (2012).

10. Grimmer, J. & Stewart, B. M. Text as Data: The Promise and Pitfalls of Automatic Content Analysis Methods for Political Texts. *Polit. analysis* **21**, 267–297 (2013).

11. Grimmer, J., Roberts, M. E. & Stewart, B. M. *Text as Data: A New Framework for Machine Learning and the Social Sciences* (Princeton University Press, 2022).

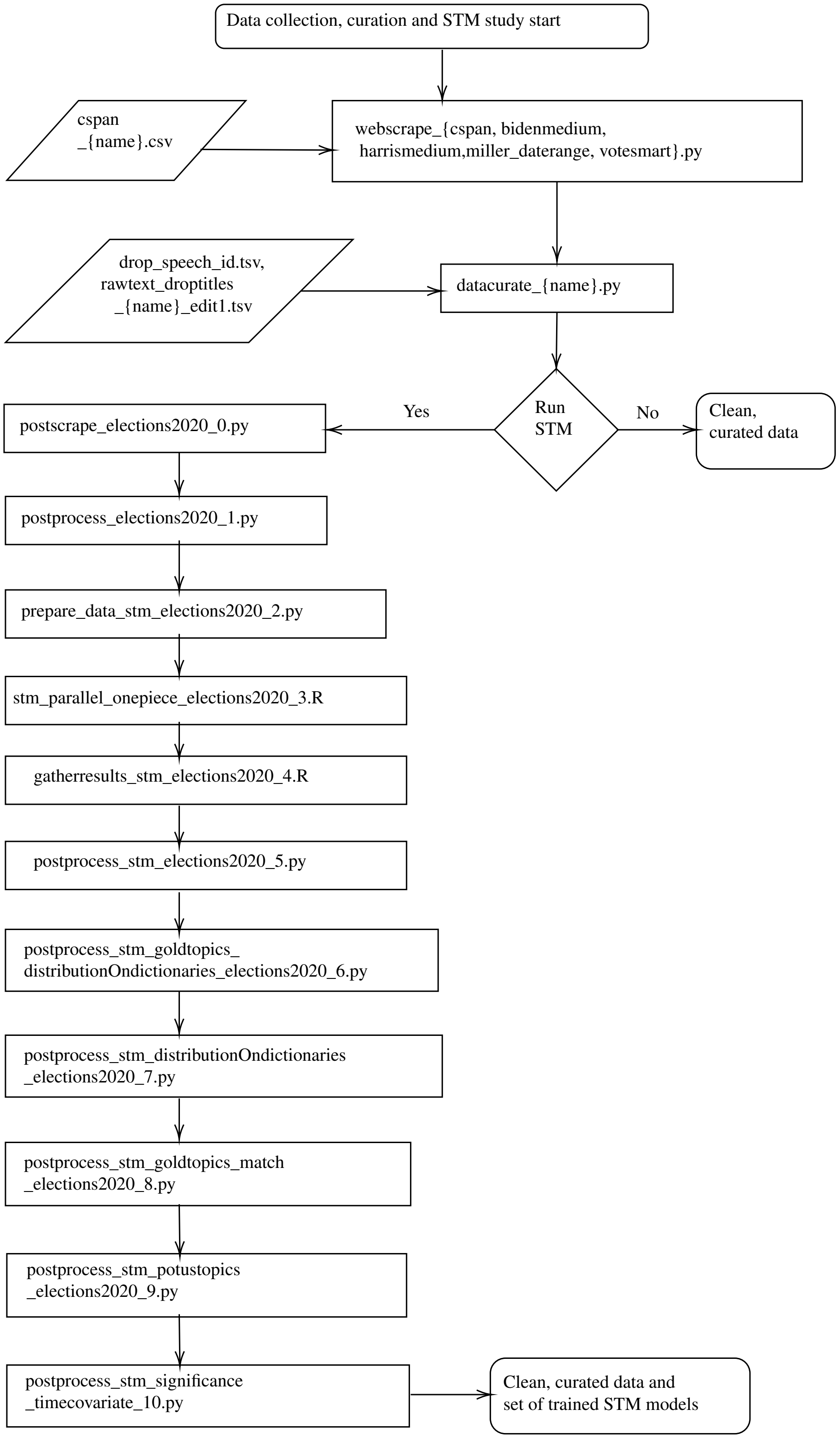

Figure S1. Overview of the code scripts and the execution order to collect, curate the dataset and run the STM case study. In the {name} fields, the corresponding candidate's name should be introduced.

Algorithm 1: Discovery and label assignment of campaign topics.

Input : i) Best achieved topic model for a given time period in our dataset M comprised of K topics $\Gamma = \{\gamma_1, \ldots, \gamma_K\}$, topic proportion per document in M, $\boldsymbol{T} \in \mathbb{R}^{|M| \times K}$ and words associated with each topic
ii) Set of clusters $\mathscr{C}$ of correlated topics for the corresponding topic model, after applying the Structural Topic Model and graph LASSO
iii) A dictionary $\mathbb{D}$ comprised of a set of J sub-dictionaries $\mathbb{D} = \{\mathbb{D}_1, \ldots, \mathbb{D}_J\}$,
iv) An annotation dictionary $\mathbb{D}^\alpha$ comprised of a set of I annotation dictionaries $\mathbb{D}^\alpha = \{\mathbb{D}_1^\alpha, \ldots, \mathbb{D}_I^\alpha\}$ and their associated names

Output : Dominant topics for the given period and their thematic labels

1. For each cluster set $c = \{\gamma_{c_1} \ldots, \gamma_{c_L}\} \in \mathscr{C}$, $c_l \in [1, K]$ comprised of $|c|$ STM-determined topics, compute the median $\hat{m}_c$ of the proportions of all STM topics $\gamma_{c_l} \in c$, in all documents M.

2. Determine the dominant topics $\tilde{\mathscr{C}} \subseteq \mathscr{C}$ as those cluster sets whose median proportion in the documents M is in the 6th decile of all cluster sets medians, as computed over all elements of $\mathscr{C}$ in Step 1:

$$\tilde{\mathscr{C}} = \{c | \hat{m}_c \in \mathscr{Q}^{\text{thresh}}\},$$

   where thresh= 0.6 and $\mathscr{Q}^{\text{thresh}}$ denotes the 6th decile of $\{\hat{m}_c\}_{c=1}^{|C|}$.

3. For each dominant topic $\tilde{c} \in \tilde{\mathscr{C}}$, keep the documents $\mathscr{D}_{\tilde{c}}$ that contain $\tilde{c}$ in a proportion of at least 80%. If no documents achieve this percentage, keep the document with the highest percentage in terms of content for the topic:

$$\mathscr{D}_{\tilde{c}} = \{d | T_{d,p} > 0.8 \text{ or } T_{d,p} = \max(T_{:,p}) \leq 0.8\}, \; \forall p \in \tilde{c}.$$

   Extract the metalabels or the summaries (depending on document source) for these documents, which are considered representative for $\tilde{c}$.

4. For each $d \in \mathscr{D}_{\tilde{c}}$, and for each dominant topic $\tilde{c}$, collect the words that are in the metalabels/summary per topic $\tilde{c}$, and concatenate them with the list of words that are associated with topic $\tilde{c}$, as determined by the STM, to produce set $W_{\tilde{c}}$.

5. For each $\tilde{c} \in \tilde{\mathscr{C}}$, construct dictionary proportion vector $\boldsymbol{p}_{\tilde{c}}^{\mathbb{D}} \in \mathbb{R}^J$, where:

$$p_{\tilde{c},j}^{\mathbb{D}} = \frac{\text{overlap of } W_{\tilde{c}} \text{ with sub-dictionary } \mathbb{D}^j}{\text{overlap of } W_{\tilde{c}} \text{ with full dictionary } \mathbb{D}} = \frac{|W_{\tilde{c}} \cap \mathbb{D}^j|}{\sum_{m=1}^{\mathbb{D}} |W_{\tilde{c}} \cap \mathbb{D}^m|}.$$

6. For each $\mathbb{D}_i^\alpha \in \mathbb{D}^\alpha$, construct the annotation dictionary proportion vector $\boldsymbol{\alpha}_i^{\mathbb{D}} \in \mathbb{R}^J$, where:

$$\alpha_{i,j}^{\mathbb{D}} = \frac{\text{overlap of annotation dictionary } \mathbb{D}_i^\alpha \text{ with sub-dictionary } \mathbb{D}^j}{\text{overlap of annotation dictionary } \mathbb{D}_i^\alpha \text{ with full dictionary } \mathbb{D}} = \frac{|\mathbb{D}_i^\alpha \cap \mathbb{D}^j|}{\sum_{m=1}^{\mathbb{D}} |\mathbb{D}_i^\alpha \cap \mathbb{D}^m|}.$$

7. Compute the similarity of each dominant topic $\tilde{c}$ with the theme of each annotation dictionary $\{\mathbb{D}^\alpha\}_1^I$ using their cosine distances:

$$s(\boldsymbol{p}_{\tilde{c}}^{\mathbb{D}}, \boldsymbol{\alpha}_i^{\mathbb{D}}) = 1 - \frac{(\boldsymbol{p}_{\tilde{c}}^{\mathbb{D}})^T \boldsymbol{\alpha}_i^{\mathbb{D}}}{\|\boldsymbol{p}_{\tilde{c}}^{\mathbb{D}}\|_2 \|\boldsymbol{\alpha}_i^{\mathbb{D}}\|_2}, \; i = 1, \ldots, I.$$

8. Assign to dominant topic $\tilde{c}$ the name of the dictionary $\mathbb{D}_{\hat{i}}^\alpha$ such that $\hat{i} = \text{argmax}_i \left(s(\boldsymbol{p}_{\tilde{c}}^{\mathbb{D}}, \boldsymbol{\alpha}_i^{\mathbb{D}}) \right)$, $i = 1, \ldots, I$.

12. Roberts, M. E., Stewart, B. M. & Tingley, D. Navigating the Local Modes of Big Data: The Case of Topic Models. *Comput. social science* **51** (2016).

13. Zirn, C. & Stuckenschmidt, H. Multidimensional topic analysis in political texts. *Data & Knowl. Eng.* **90**, 38–53 (2014).

14. Béchara, Hannah and Herzog, Alexander and Jankin, Slava and John, Peter. Transfer learning for topic labeling: Analysis of the UK House of Commons speeches 1935–2014. *Res. & Polit.* **8** (2021).

15. Osnabrügge, M., Ash, E. & Morelli, M. Cross-Domain Topic Classification for Political Texts. *Polit. Analysis* **31**, 59–80 (2023).

16. Budge, I., Klingemann, H., Volkens, A., Bara, J. & Tanenbaum, E. *Mapping Policy Preferences. Estimates for Parties, Electors, and Governments 1945-1998*, vol. 1 (Oxford University Press, 2001).

17. Klingemann, H.-D., Volkens, A., Bara, J., Budge, I. & McDonald, M. D. *Mapping Policy Preferences II: Estimates for Parties, Electors, and Governments in Eastern Europe, European Union, and OECD 1990-2003*, vol. 2 (Oxford University Press, 2006).

18. Merz, N., Regel, S. & Lewandowski, J. The Manifesto Corpus: A new resource for research on political parties and quantitative text analysis. *Res. & Polit.* **3** (2016).
